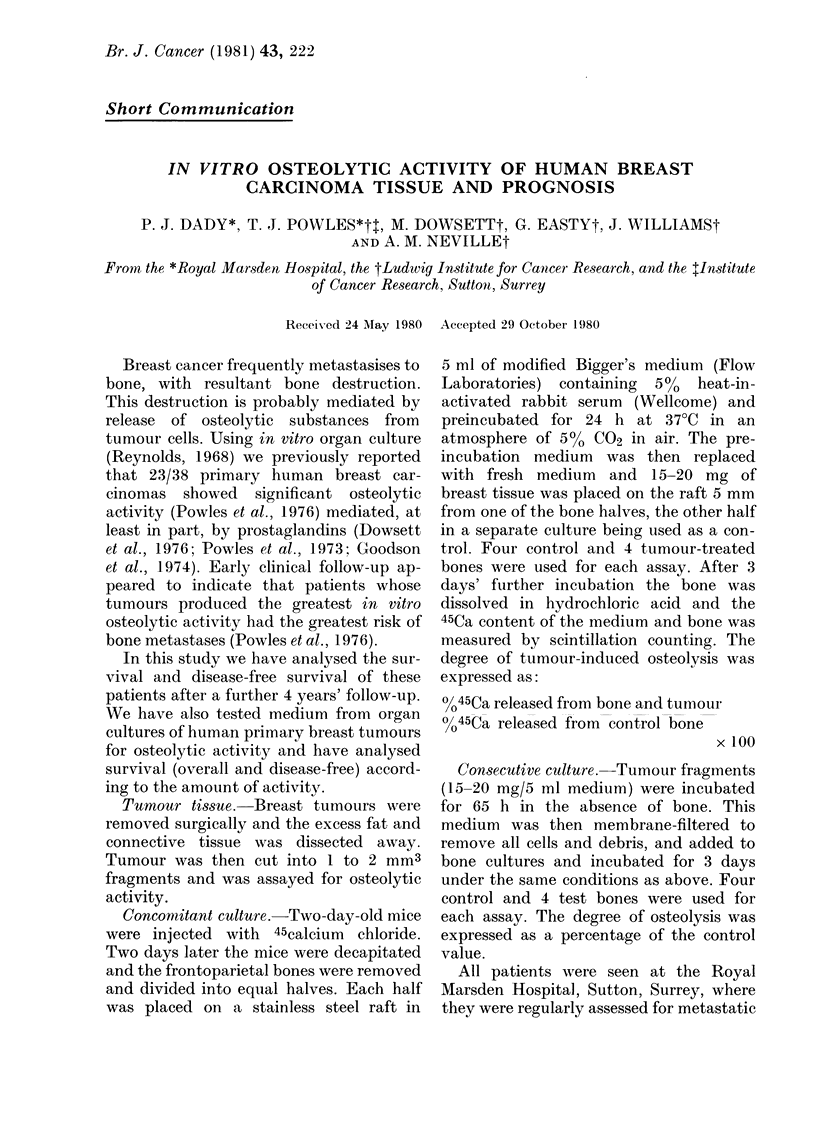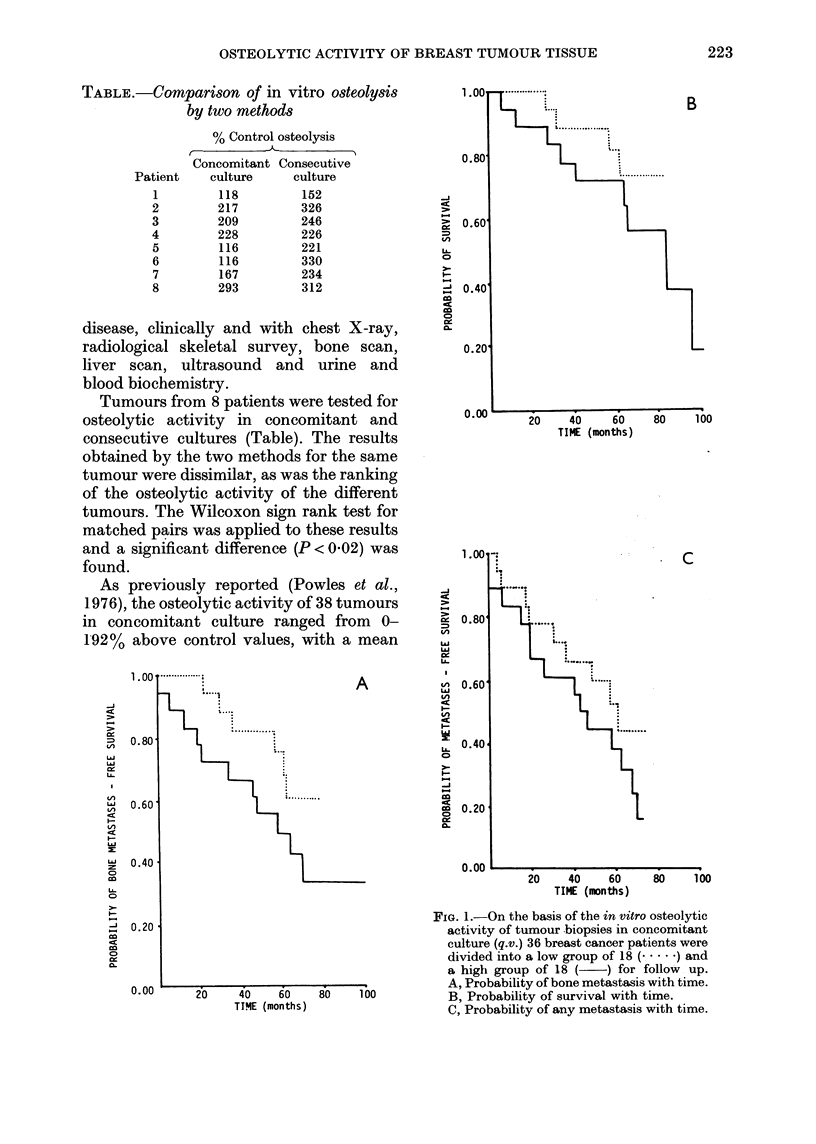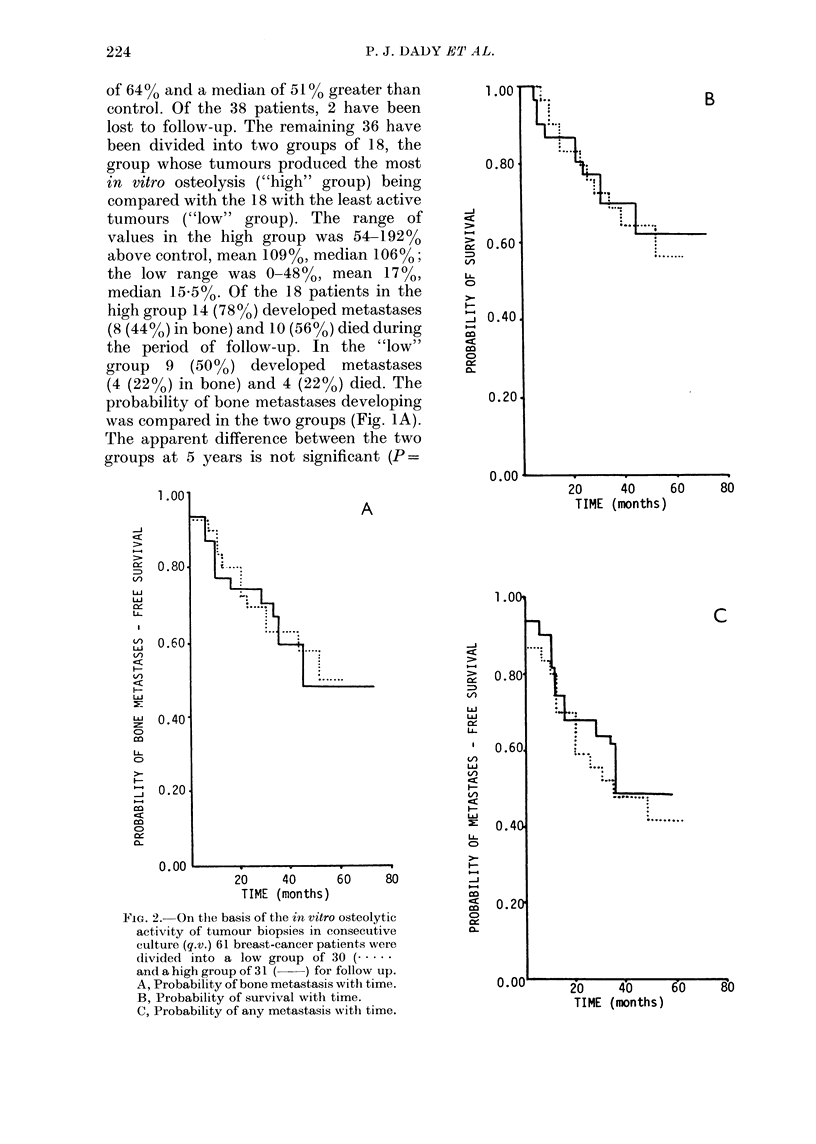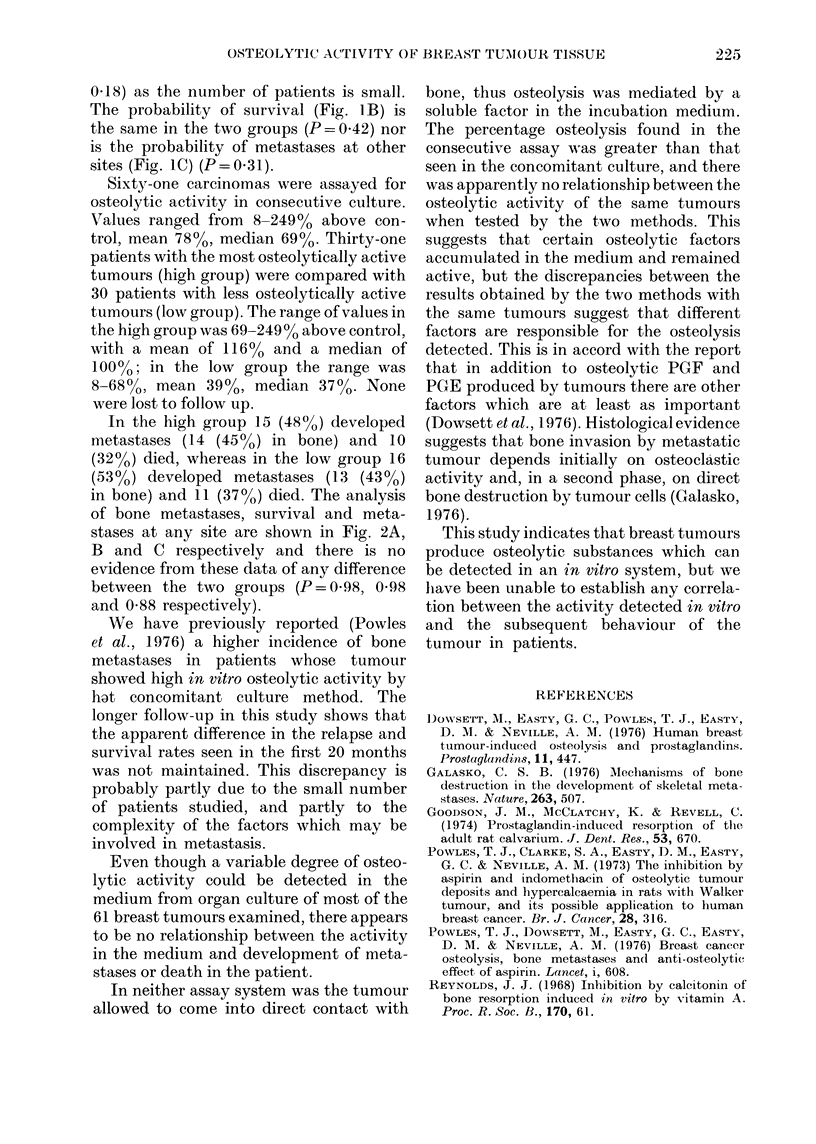# In vitro osteolytic activity of human breast carcinoma tissue and prognosis.

**DOI:** 10.1038/bjc.1981.31

**Published:** 1981-02

**Authors:** P. J. Dady, T. J. Powles, M. Dowsett, G. Easty, J. Williams, A. M. Neville


*Br. J. Cancer* (1981) **43**, 222

***Short Communication***

### *IN VITRO* OSTEOLYTIC ACTIVITY OF HUMAN BREAST CARCINOMA TISSUE AND PROGNOSIS

P. J. DADY*, T. J. POWLES*†‡, M. DOWSETT†, G. EASTY†, J. WILLIAMS† AND A. M. NEVILLE†

*From the *Royal Marsden Hospital, the †Ludwig Institute for Cancer Research, and the ‡Institute of Cancer Research, Sutton, Surrey*

Received 24 May 1980  Accepted 29 October 1980

Breast cancer frequently metastasises to bone, with resultant bone destruction. This destruction is probably mediated by release of osteolytic substances from tumour cells. Using *in vitro* organ culture (Reynolds, 1968) we previously reported that 23/38 primary human breast carcinomas showed significant osteolytic activity (Powles *et al.*, 1976) mediated, at least in part, by prostaglandins (Dowsett *et al.*, 1976; Powles *et al.*, 1973; Goodson *et al.*, 1974). Early clinical follow-up appeared to indicate that patients whose tumours produced the greatest *in vitro* osteolytic activity had the greatest risk of bone metastases (Powles *et al.*, 1976).

In this study we have analysed the survival and disease-free survival of these patients after a further 4 years' follow-up. We have also tested medium from organ cultures of human primary breast tumours for osteolytic activity and have analysed survival (overall and disease-free) according to the amount of activity.

*Tumour tissue.*—Breast tumours were removed surgically and the excess fat and connective tissue was dissected away. Tumour was then cut into 1 to 2 $mm^3$ fragments and was assayed for osteolytic activity.

*Concomitant culture.*—Two-day-old mice were injected with $^{45}$calcium chloride. Two days later the mice were decapitated and the frontoparietal bones were removed and divided into equal halves. Each half was placed on a stainless steel raft in 5 ml of modified Bigger's medium (Flow Laboratories) containing 5% heat-inactivated rabbit serum (Wellcome) and preincubated for 24 h at 37°C in an atmosphere of 5% $CO_2$ in air. The preincubation medium was then replaced with fresh medium and 15–20 mg of breast tissue was placed on the raft 5 mm from one of the bone halves, the other half in a separate culture being used as a control. Four control and 4 tumour-treated bones were used for each assay. After 3 days' further incubation the bone was dissolved in hydrochloric acid and the $^{45}$Ca content of the medium and bone was measured by scintillation counting. The degree of tumour-induced osteolysis was expressed as:

$$\frac{\%\,^{45}\text{Ca released from bone and tumour}}{\%\,^{45}\text{Ca released from control bone}} \times 100$$

*Consecutive culture.*—Tumour fragments (15–20 mg/5 ml medium) were incubated for 65 h in the absence of bone. This medium was then membrane-filtered to remove all cells and debris, and added to bone cultures and incubated for 3 days under the same conditions as above. Four control and 4 test bones were used for each assay. The degree of osteolysis was expressed as a percentage of the control value.

All patients were seen at the Royal Marsden Hospital, Sutton, Surrey, where they were regularly assessed for metastatic

TABLE.—*Comparison of* in vitro *osteolysis by two methods*

| Patient | % Control osteolysis: Concomitant culture | Consecutive culture |
|---|---|---|
| 1 | 118 | 152 |
| 2 | 217 | 326 |
| 3 | 209 | 246 |
| 4 | 228 | 226 |
| 5 | 116 | 221 |
| 6 | 116 | 330 |
| 7 | 167 | 234 |
| 8 | 293 | 312 |

disease, clinically and with chest X-ray, radiological skeletal survey, bone scan, liver scan, ultrasound and urine and blood biochemistry.

Tumours from 8 patients were tested for osteolytic activity in concomitant and consecutive cultures (Table). The results obtained by the two methods for the same tumour were dissimilar, as was the ranking of the osteolytic activity of the different tumours. The Wilcoxon sign rank test for matched pairs was applied to these results and a significant difference ($P < 0{\cdot}02$) was found.

As previously reported (Powles *et al.*, 1976), the osteolytic activity of 38 tumours in concomitant culture ranged from 0–192% above control values, with a mean

FIG. 1.—On the basis of the *in vitro* osteolytic activity of tumour biopsies in concomitant culture (*q.v.*) 36 breast cancer patients were divided into a low group of 18 (· · · · ·) and a high group of 18 (———) for follow up. A, Probability of bone metastasis with time. B, Probability of survival with time. C, Probability of any metastasis with time.

of 64% and a median of 51% greater than control. Of the 38 patients, 2 have been lost to follow-up. The remaining 36 have been divided into two groups of 18, the group whose tumours produced the most *in vitro* osteolysis ("high" group) being compared with the 18 with the least active tumours ("low" group). The range of values in the high group was 54–192% above control, mean 109%, median 106%; the low range was 0–48%, mean 17%, median 15·5%. Of the 18 patients in the high group 14 (78%) developed metastases (8 (44%) in bone) and 10 (56%) died during the period of follow-up. In the "low" group 9 (50%) developed metastases (4 (22%) in bone) and 4 (22%) died. The probability of bone metastases developing was compared in the two groups (Fig. 1A). The apparent difference between the two groups at 5 years is not significant ($P=$

FIG. 2.—On the basis of the *in vitro* osteolytic activity of tumour biopsies in consecutive culture (*q.v.*) 61 breast-cancer patients were divided into a low group of 30 (· · · · · and a high group of 31 (——) for follow up. A, Probability of bone metastasis with time. B, Probability of survival with time. C, Probability of any metastasis with time.

0·18) as the number of patients is small. The probability of survival (Fig. 1B) is the same in the two groups ($P = 0{\cdot}42$) nor is the probability of metastases at other sites (Fig. 1C) ($P = 0{\cdot}31$).

Sixty-one carcinomas were assayed for osteolytic activity in consecutive culture. Values ranged from 8–249% above control, mean 78%, median 69%. Thirty-one patients with the most osteolytically active tumours (high group) were compared with 30 patients with less osteolytically active tumours (low group). The range of values in the high group was 69–249% above control, with a mean of 116% and a median of 100%; in the low group the range was 8–68%, mean 39%, median 37%. None were lost to follow up.

In the high group 15 (48%) developed metastases (14 (45%) in bone) and 10 (32%) died, whereas in the low group 16 (53%) developed metastases (13 (43%) in bone) and 11 (37%) died. The analysis of bone metastases, survival and metastases at any site are shown in Fig. 2A, B and C respectively and there is no evidence from these data of any difference between the two groups ($P = 0{\cdot}98$, 0·98 and 0·88 respectively).

We have previously reported (Powles *et al.*, 1976) a higher incidence of bone metastases in patients whose tumour showed high *in vitro* osteolytic activity by hət concomitant culture method. The longer follow-up in this study shows that the apparent difference in the relapse and survival rates seen in the first 20 months was not maintained. This discrepancy is probably partly due to the small number of patients studied, and partly to the complexity of the factors which may be involved in metastasis.

Even though a variable degree of osteolytic activity could be detected in the medium from organ culture of most of the 61 breast tumours examined, there appears to be no relationship between the activity in the medium and development of metastases or death in the patient.

In neither assay system was the tumour allowed to come into direct contact with bone, thus osteolysis was mediated by a soluble factor in the incubation medium. The percentage osteolysis found in the consecutive assay was greater than that seen in the concomitant culture, and there was apparently no relationship between the osteolytic activity of the same tumours when tested by the two methods. This suggests that certain osteolytic factors accumulated in the medium and remained active, but the discrepancies between the results obtained by the two methods with the same tumours suggest that different factors are responsible for the osteolysis detected. This is in accord with the report that in addition to osteolytic PGF and PGE produced by tumours there are other factors which are at least as important (Dowsett *et al.*, 1976). Histological evidence suggests that bone invasion by metastatic tumour depends initially on osteoclastic activity and, in a second phase, on direct bone destruction by tumour cells (Galasko, 1976).

This study indicates that breast tumours produce osteolytic substances which can be detected in an *in vitro* system, but we have been unable to establish any correlation between the activity detected *in vitro* and the subsequent behaviour of the tumour in patients.

### REFERENCES

Dowsett, M., Easty, G. C., Powles, T. J., Easty, D. M. & Neville, A. M. (1976) Human breast tumour-induced osteolysis and prostaglandins. *Prostaglandins*, **11**, 447.

Galasko, C. S. B. (1976) Mechanisms of bone destruction in the development of skeletal metastases. *Nature*, **263**, 507.

Goodson, J. M., McClatchy, K. & Revell, C. (1974) Prostaglandin-induced resorption of the adult rat calvarium. *J. Dent. Res.*, **53**, 670.

Powles, T. J., Clarke, S. A., Easty, D. M., Easty, G. C. & Neville, A. M. (1973) The inhibition by aspirin and indomethacin of osteolytic tumour deposits and hypercalcaemia in rats with Walker tumour, and its possible application to human breast cancer. *Br. J. Cancer*, **28**, 316.

Powles, T. J., Dowsett, M., Easty, G. C., Easty, D. M. & Neville, A. M. (1976) Breast cancer osteolysis, bone metastases and anti-osteolytic effect of aspirin. *Lancet*, i, 608.

Reynolds, J. J. (1968) Inhibition by calcitonin of bone resorption induced *in vitro* by vitamin A. *Proc. R. Soc. B.*, **170**, 61.